# Alterations in Serotonin Neurotransmission in Hyperdopaminergic Rats Lacking the Dopamine Transporter

**DOI:** 10.3390/biomedicines11112881

**Published:** 2023-10-24

**Authors:** Dmitrii S. Traktirov, Ilya R. Nazarov, Valeria S. Artemova, Raul R. Gainetdinov, Nina S. Pestereva, Marina N. Karpenko

**Affiliations:** 1 Department of Physiology (Pavlov’s), Institute of Experimental Medicine, 197022 St. Petersburg, Russiamnkarpenko@mail.ru (M.N.K.); 2 Faculty of Biology, St. Petersburg State University, 199034 St. Petersburg, Russia; 3 Institute of Biomedical Systems and Biotechnologies, Peter the Great St. Petersburg Polytechnic University, 195251 St. Petersburg, Russia; 4 Institute of Translational Biomedicine, St. Petersburg University Hospital, St. Petersburg State University, 199034 St. Petersburg, Russia; gainetdinov.raul@gmail.com

**Keywords:** DAT-KO, dopamine, serotonin, ADHD, addiction, schizophrenia, depression

## Abstract

Biogenic amines dopamine (DA) and serotonin (5-HT) are among the most significant monoaminergic neurotransmitters in the central nervous system (CNS). Separately, the physiological roles of DA and 5-HT have been studied in detail, and progress has been made in understanding their roles in normal and various pathological conditions (Parkinson’s disease, schizophrenia, addiction, depression, etc.). In this article we showed that knockout of the gene encoding DAT leads not only to a profound dysregulation of dopamine neurotransmission in the striatum but also in the midbrain, prefrontal cortex, hippocampus, medulla oblongata and spinal cord. Furthermore, significant changes were observed in the production of mRNA of enzymes of monoamine metabolism, as well as to a notable alteration in the tissue level of serotonin, most clearly manifested in the cerebellum and the spinal cord. The observed region-specific changes in the tissue levels of serotonin and in the expression of dopamine and serotonergic metabolism enzymes in rats with an excess of dopamine can indicate important consequences for the pharmacotherapy of drugs that modulate the dopaminergic system. The drugs that affect the dopaminergic system could potently affect the serotonergic system, and this fact is important to consider when predicting their possible therapeutic or side effects.

## 1. Introduction

Dopamine (DA) and serotonin (5-HT) are important monoaminergic neurotransmitters in the central nervous system (CNS). Brain dopamine, predominantly synthesized in the substantia nigra pars compacta (SNpc), the ventral tegmental area (VTA), and the arcuate nucleus of the hypothalamus, mainly regulates motor control, reward-based learning, arousal, addiction, activeness, motivation, cognitive function and hormonal regulation [1,2,3]. Serotonergic pathways originating from dorsal and medial raphe nuclei innervate a variety of cortical and subcortical structures and determine the serotonin involvement in psychomotor inhibition, the regulation of emotions and mood, cognition and adaptation to stressors [4,5]. Separately, the physiological roles of DA and 5-HT have been studied in detail, and progress has been made in understanding their roles in normal and various pathological conditions (Parkinson’s disease, schizophrenia, addiction, depression, etc.) [6,7,8]. However, there are also several reports indicating that these systems can closely interact through variety of neurochemical mechanisms [9,10,11]. Thus, functional disturbances in one of these systems may lead to alterations in the other system. For example, DA deficiency in Parkinson’s disease (PD) leads to the development of mental disorders—anhedonia and loss of motivation—that are likely associated with not only dopaminergic dysfunction but also with a violation in the serotonin system [12]. At the same time, the canonical PD treatment with levodopa (L-dopa) leads to the reduction of both motor and non-motor symptoms of PD [13,14]. Dopamine-5-HT interaction may contribute to the established role of DA in the regulation of mood and behavioral conditions. The reverse is also true: 5-HT may play an important role in regulating DA-dependent behavior [15,16,17]. Besides functional interactions between the dopaminergic and serotoninergic systems, their anatomical [18,19] and neurochemical [20,21] connections were shown. For example, it has been reported that 5-HT transporters (SERT) at certain conditions can uptake part of DA from the extracellular space, and vice versa, DA transporters (DAT) may potentially uptake 5-HT [20,22]. It should be noted, however, that no alterations in DA clearance in the striatum were observed in mice and rats lacking the dopamine transporter (*DAT knockout*, *DAT-KO*) treated with serotonin reuptake inhibitor (SSRI) fluoxetine [23,24]. Therefore, animals with altered DA neurotransmission could be very useful for studying the interaction of DA and 5-HT systems. Partially, this approach has already been tested in mice lacking the dopamine transporter (*DAT-KO* mice) [25]. *DAT-KO* mice are characterized by an elevated extracellular levels of DA and reduced levels of D1/D2 dopamine receptors [23,26]. These animals develop perseverative, compulsive, stereotypical, and hyperactive behaviors [27,28], which may be indicative also of 5-HT metabolism dysfunction. Indeed, DAT-KO mice show an increase in tissue 5-HT levels in the hippocampus and a decrease in 5-HT in the striatum [23,28]. Recently developed DAT-KO rats are also characterized by a distinct set of behavioral disorders; they demonstrate pronounced hyperactivity, stereotypy, deficit in working memory and less propensity to develop obsessive behaviors [24,29]. Consequently, the functioning of the 5-HT-ergic system in DAT-KO rats could be expected to be altered throughout the brain, but this has not been investigated in full detail yet. Thus, the aim of this study was to investigate changes in the serotoninergic system, such as area-dependent changes in serotonin content and serotonin turnover rates, and changes in the mRNA content of monoamines metabolism enzymes in DAT-KO rats.

## 2. Material and Methods

### 2.1. Animals

The study was performed on *DAT-KO* (*n* = 13) and DAT-WT (*n* = 10) rats obtained from the vivarium of the Institute of Translational Biomedicine, St. Petersburg State University, St. Petersburg, Russia. The animals were housed in galvanized polypropylene cages of 5 rats each in a room with controlled conditions (24 ± 1 °C for temperature, 45–65% humidity, and 12 h light/12 h dark cycle). In the period of the experiment, the pelleted rat chow and water were available ad libitum. After the start of the experiment, no animals were excluded.

### 2.2. Sample Collection

Rats were euthanized with Zoletil (15 mg/kg), after which they were decapitated with a guillotine (OpenScience AE1601, RPC OpenScience Ltd., Krasnogorsk, Russia). Tissue samples were taken according to the brain atlas [30]. Collected tissues were immediately frozen and stored at −80 °C until further analysis.

### 2.3. Genotyping

Genotyping was performed after the molecular experiments. DNA was extracted from fragments of tail tissue. Genotyping was performed by DAT gene DNA amplification with PCR followed by restriction enzyme BtsIMutI (New England Biolabs, Ipswich, Massachusetts, USA) digestion and electrophoretic separation. The details of the method are described in [24].

### 2.4. Determination of Monoamines

For the quantification of 5-HT, its metabolite 5-hydroxyindoleacetic acid (5-HIAA), and dopamine (DA) and its metabolites 3,4-dihydroxyphenylacetic acid (DOPAC) and homovanillic acid (HVA), high-performance liquid chromatography (HPLC) system was used. First, brain tissue was homogenized in 0.1 M perchloric acid, then centrifuged at 9000× *g* and the supernatant was taken for analysis. The HPLC analysis was performed on a C18 reverse-phase column BDS Hypersil (250 × 4.6 mm, particle size 5 μm) under isocratic conditions with electrochemical detection. The mobile phase consisted of a 75 mM phosphate buffer containing 2 mM citrate acid, 0.1 mM octanesulfonic acid, and 5% (*v*/*v*) acetonitrile (pH 3.1). Monoamine content was corrected for total protein concentration of the final sample, as assessed by the Pierce bicinchoninic acid (BCA) assay according to the manufacturer’s instructions (Thermo Fisher Scientific, Waltham, Massachusetts, USA) and was expressed as ng/mg of total protein content using an external calibration curve.

### 2.5. RNA Isolation

Total RNA was isolated from brain regions using TRIzol Reagent (Invitrogen, Thermo Fisher Scientific, Waltham, Massachusetts, USA). RNA concentration was measured using a NanoDrop 2000 spectrophotometer (Thermo Scientific, Waltham, Massachusetts, USA) following the standard procedure. The purity of RNA samples was verified by confirming that each had an optical density ratio A260/A280 > 1.8. To verify the integrity of the samples, the 18S/28S RNA ratio was analyzed after electrophoresis in 1.4% agarose gel.

### 2.6. cDNA Synthesis and Real-Time RT–PCR

Two μg of total RNA was used for cDNA synthesis using high-capacity DNA reverse transcription kits (Applied Biosystems, Thermo Scientific, Waltham, Massachusetts, USA). Quantitative real time RT–PCR was performed using Evrogen 5× qPCR mix–HS SYBR (CFX Bio-Rad, Hercules, California, USA). Primers were designed with the Primer-BLAST tool (NCBI, Bethesda, Maryland, USA, https://www.ncbi.nlm.nih.gov/tools/primer-blast/ accessed on 18 October 2023); the primer sequences and sizes of PCR products are presented in the Table 1. The PCR parameters were as follows: initial denaturation (95 °C for 15 min); 40 cycles of denaturation, annealing, and extension (95 °C for 15 s, X °C for 30 s, and 72 °C for 5 s; for X see Table 1); and the melting curve (starting at 65 °C and gradually increasing to 95 °C). 18S mRNA was used as an internal control. The delta–delta CT method was used to determine the fold increase of genes relative to the control group. The results are given as bar charts. Each value was combined from 3 independent PCR replicates for each cDNA sample. The data were normalized to the control group.

### 2.7. Statistical Analysis

Statistical analysis was conducted using Statistica 12.0 (StatSoft, Dell, Round Rock, Texas, USA). The normality of the distribution was verified by the Shapiro–Wilk test. All data are expressed as the means ± SEM. Statistical differences were tested with the *t*-test. *p* < 0.05 was considered statistically significant.

## 3. Results

### 3.1. DA, 5-HT and Their Metabolites in the Different Regions of CNS of DAT-KO Rats

We have analyzed the content of DA, 5-HT and their metabolites in the striatum, prefrontal cortex, hippocampus, medulla oblongata, cerebellum and cervical spinal cord of *DAT-KO* rats (Table 2). Significant differences were detected for 5-HT and HIAA for most of the analyzed structures, and the differences in levels of DA and its metabolites in *DAT-WT* and *DAT-KO* rats were observed in every structure except the cerebellum. It turned out that in the striatum, medulla oblongata and spinal cord of *DAT-KO* rats DA levels were 8.5, 3.7 and 5.0 times lower, respectively, as compared with the control group (the corresponding *p*-values are given in the Table 2). DA levels in the cortex and hippocampus of *DAT-KO* rats were 2.3 times higher as compared with the control group. Also, we observed an increase in the content of DA metabolites in the striatum and cortex of *DAT-KO* animals: these rats had 2.3 times higher levels of DOPAC and 3.6 times higher levels of HVA in striatum; in the cortex, the DOPAC content was 2.5 times higher, compared with the control, with HVA levels being unchanged. In medulla oblongata, the content of DOPAC was three times lower compared with the control with unchanged HVA compared with the control. The 5-HT level was decreased in the cortex, medulla oblongata, cerebellum and spinal cord of *DAT-KO* rats by 2.4, 29.2, 13.5 and 3.3 times, respectively, compared with the control group. A decrease in the 5-HT content was accompanied with a decrease in the content of its metabolite 5-HIAA in the medulla oblongata and cerebellum by 3.4 and 2.0 times, respectively. We observed an increase in 5-HIAA content in the hippocampus by 1.1 times, relative to the control group, with the not significantly changed levels of 5-HT. In the cortex and spinal cord, where a decrease in 5-HT level was observed, no significant change in the 5-HIAA content was found in comparison with the control group. The midbrain DA level was 2 times lower, and the midbrain 5-HT level was 1.9 times lower, compared with the control group.

### 3.2. Turnover Rates of DA and 5-HT in the Different Regions of DAT-KO Rats

DOPAC/DA and HVA/DA ratios were used to assess intracellular and extracellular DA turnover rates, respectively, because DOPAC is a major intracellular DA metabolite while HVA—a major extracellular DA metabolite [31]. It was shown that intracellular DA turnover rates in the striatum and spinal cord of *DAT-KO* rats were 20 and 2 times lower in comparison to the control group, respectively (the corresponding *p*-values are given in the Table 3), while extracellular DA turnover rate was intensified only in the striatum (38 times higher in *DAT-KO* rats). On the contrary, the intracellular DA turnover rates were 2.0 and 1.5 times lower in the hippocampus and medulla oblongata of *DAT-KO* rats relative to the control group, respectively. The extracellular DA turnover rate in the hippocampus could not be determined, and it did not differ between groups in the medulla oblongata. The intracellular DA turnover rate in the prefrontal cortex of *DAT-KO* rats did not differ from the control animals, and the extracellular DA turnover rate was four times lower in the same brain region of *DAT-KO* rats.

We also used the 5-HIAA/5-HT conversion ratio as an index of 5-HT turnover. It turned out that there were 5.0 and 4.4-fold increases in the turnover rates of 5-HT in the cerebellum and spinal cord of *DAT-KO* rats relative to the control, respectively. The turnover rates of 5-HT in the cortex and hippocampus of *DAT-KO* animals were comparable to the values in the control group.

### 3.3. mRNA Expression of the Main Enzymes Involved in Monoamine Metabolism in Different Regions of DAT-KO Rats

We determined mRNA expression of the MAO-A, MAO-B and COMT genes in striatum, cortex, hippocampus, medulla oblongata, cerebellum and spinal cord of *DAT-KO* rats (Table 4). The 2.5- and 5-times lower expressions of MAO-A and MAO-B mRNA, respectively, were discovered in the striatum of *DAT-KO* rats compared with the control group (the corresponding *p*-values are given in the Table 4). The same differences were observed in medulla oblongata: the mRNA expression of MAO-A was 12.0 times lower, the mRNA expression of MAO-B—10.0 times lower and the mRNA expression of COMT—5.0 times lower in *DAT-KO* rats compared with the control group. Conversely, the following mRNA expression was detected to be higher in *DAT-KO* rats: 2.1-fold increase in MAO-A mRNA expression in the prefrontal cortex, 2.0-fold increase in MAO-B mRNA expression in the prefrontal cortex, 5.0-fold increase in COMT mRNA expression in the cerebellum. There was a 1.2-fold decrease in mRNA expression of MAO-A, 13-fold increase in mRNA expression of MAO-B, and 10.8-fold increase in mRNA expression of COMT in the spinal cord of *DAT-KO* rats compared with the control group. Also, MAO-B mRNA expression in the hippocampus of *DAT-KO* rats was 2.2 times higher in comparison to the control group.

## 4. Discussion

Here, we demonstrate that the lack of DAT causes dramatic alterations in dopaminergic neurochemical parameters not only in the striatum, where it has been extensively investigated previously, but also in other brain areas. Furthermore, significant changes in the serotonin system and mRNA of degradative enzymes were found in the most brain areas analyzed.

The nigrostriatal pathway is one of the most studied and functionally important dopaminergic pathways. It connects the compact zone of the substantia nigra and the dorsal striatum. The levels of both dopamine and serotonin were 2- and 1.9-times lower in the midbrain of *DAT-KO* rats compared with the control (Table 2). A similar decreasing trend was observed in the striatum, where in the KO group there had a more dramatic 8.6-fold reduction in the DA content. The results are consistent with previous neurochemical investigations in the striatum both in *DAT-KO* mice and rats. It has been reported that the levels of tissue DA in the striatum of *DAT-KO* mice and rats are lower than in the control group by 20 and 13 times, respectively, while the levels of dopamine metabolites—DOPAC and HVA—are higher than in the controls [23,24]. At the same time, the levels of extracellular dopamine in the striatum of *DAT-KO* mice and rats measured by the microdialysis technique were five and seven times higher than in WT controls [23,24]. While a 25% decrease in the expression of mRNA of Tyrosine hydroxylase (TH) occurred in the ventral midbrain of *DAT-KO* mice, with the level of TH protein expression in this structure reduced by 35%, a more dramatic 90% decrease of the level of TH protein expression was observed in the striatum [32]. However, the decreased TH protein level cannot explain dramatic reduction of DA striatal level in *DAT-KO* rats since TH activity in fact increased two-fold [33]. Rather, it indicates important role of DAT-mediated re-uptake in supporting intraneuronal DA stores [23,34]. In fact, remaining intracellular DA in animals lacking DAT is becoming totally dependent on ongoing synthesis and inhibition of TH essentially eliminates striatal tissue DA in mutants while decreases it only by 50% in WT mice and rats [35,36]. The decrease of DA in striatum was accompanied by an increase in the level of DA metabolites: 2.4-fold increased DOPAC and 3.6-fold increased HVA. Thus, the conversion ratios were 20 and 38 times higher for DOPAC/DA and HVA/DA, respectively. Consistent with previous observations [23,24] striatal 5-HT tissue levels were decreased. It is likely that the reason for the altered conversion ratios of DA may be related to alterations in the functioning of enzymes that metabolize monoamines.

MAO-A, MAO-B and COMT are main enzymes involved in the degradation process for various monoamines, including DA and 5-HT [37,38]. In this study, we analyzed changes in mRNA expression of these enzymes by using RT-PCR. We have shown a significant decrease in the mRNA expression of MAO-A, MAO-B, and COMT in the medulla oblongata of DAT-KO rats. A significant decrease in the mRNA expression of MAO-A (2.5 times) and MAO-B (5 times) was also observed in the striatum of *DAT-KO* rats (Table 4). These may be the result of adaptive processes: the tissue level of DA decreases and extracellular increases due to elimination of DAT, which may affect the expression of mRNA of enzymes that metabolize DA. It should be noted also that in previous studies in DAT-KO mice, no alteration was found in striatal COMT activity, but MAO activity was decreased eight-fold [33] with clearance of striatal extracellular DA being not affected by MAO or COMT inhibition both in mice and rats. However, the exact mechanism underlying the decrease of mRNA expression of enzymes remains unclear. It can be assumed that post-translational modifications of MAO-A and MAO-B proteins may lead to alterations in the stability and/or enzymatic activity of these proteins [39].

Similar remarkable alterations in dopamine neurochemistry were found also in the majority of other brain structures that were not investigated previously. The mesocortical pathway connects the ventral tegmentum of the midbrain to the frontal lobe of the cerebral cortex, particularly the prefrontal cortex. This pathway is essential for normal cognitive functioning, and is involved in the regulation of motivational and emotional responses. We observed 2.5- and 2.3-fold increased levels of DA and DOPAC in the prefrontal cortex of DAT-KO rats, respectively. It should be noted that tissue content of monoamines and their metabolites in the frontal cortex was not evaluated previously in DAT-KO mice or rats. The HVA/DA conversion ratio was 4 times lower, and the level of 5-HT was 2.4 times higher in this brain region (Table 3). Also, MAO-A and MAO-B mRNA expression were 2.1 and 2 times higher in the prefrontal cortex of DAT-KO rats compared with the control group, respectively (Table 4). These observations support the fact that the dopaminergic system demonstrates plasticity and high adaptive capacity—the levels of MAO-A and MAO-B mRNA increase in response to an increase in the level of extracellular dopamine. One possibility for the lack of change in HVA level observed in the prefrontal cortex is that the HVA/DA conversion occurs mainly outside the cell. Since DA has a high affinity not only for DAT but also for norepinephrine transporter (NET) [40] and that the density of DAT in prefrontal cortex (PFc) is relatively low [41] compared to the density of NET in PFc [42], it is possible that DA is partially captured and re-uptaken by NET, and therefore no change in HVA/DA conversion ratio is observed [43]. In fact, only a modest two-fold increase in extracellular DA levels was found in this brain area in DAT-KO mice by microdialysis technique [44], which might be not sufficient to affect significantly HVA levels.

The impairment of both working and spatial memory is usually associated with the dysregulation of the dopamine system. The role of the hippocampus in spatial learning and memory formation has been clearly established [45]. Therefore, we determined the levels of monoamines and their metabolites in the hippocampi of *DAT-KO* rats. We observed a 2.3-fold increase in the level of DA in the hippocampi of *DAT-KO* rats, and a 2-fold decrease in the DOPAC/DA conversion ratio. The levels of 5-HT and 5-HIAA remained almost unchanged. The expression of MAO-B was 2.2 times higher in knockout animals, which may be explained by adaptation to an increased content of dopamine. In addition, an increase in the NE level in the hippocampus of mice with DAT gene knockout was shown, which was associated with antisocial behavior [46], which is observed in DAT-KO animals. One of the functions of MAO-B is the ability to regulate metabolism in the adrenergic system [47]; accordingly, along with an increase in the level of NE, MAO-B expression also increased.

The most significant changes in the content of serotonin and its metabolite were observed in the cerebellum and spinal cord. In the cerebellum, the level of serotonin was 13.5 times lower and the level of 5-HIAA was 2 times lower, while the conversion of 5-HT to 5-HIAA was 5 times higher in DAT-KO rats compared to the control group. Recent studies show that, in addition to motor functions, the cerebellum regulates working memory, emotion, response timing, action planning, and attentional control [48]. Interestingly, the impairment of these functions has been found in autism spectrum disorders (ASD) and attention deficit hyperactivity disorder (ADHD) [49]. Both disorders are closely related to each other [50], overlap in genetic vulnerability [51], and share similar patterns of social impairment and increased antisocial behavior [52,53]. *DAT-KO* animals are an acknowledged model of ADHD, therefore the neurochemical changes in the cerebellum that we have identified should be taken into account in further studies of ADHD and ASD. Our results are consistent with the fact that the ability to synthesize 5-HT decreases with age in children with ASD [54,55].

The DA level was five times lower in the spinal cord of DAT-KO rats and the DOPAC level remained unchanged. DOPAC/DA conversion ratio increased two-fold. The level of 5-HT decreased 3.33-fold and the level of 5-HIAA remained unchanged, so the 5-HIAA/5-HT conversion ratio increased 4.4-fold. Expression of mRNA of enzymes that metabolize monoamines were increased in the spinal cord of DAT-KO rats: a 13-fold increase in the level of MAO-B and a 10.8-fold increase in the level of COMT. Monoamine oxidases and COMT are involved in the metabolism of not only dopamine and serotonin, but also the monoamines of the adrenergic system [47]. We expect that the levels of NE could also be altered and this topic should be explored in detail in future studies.

## 5. Highlights and Limitations

### 5.1. Highlights

1. The knockout of the gene encoding DAT in rats leads not only to a significant redistribution of dopamine in various structures of the CNS but also to pronounced changes in the level of serotonin, which are most notable in the cerebellum and the spinal cord.
2. The knockout of the gene encoding DAT leads also to alterations in the production of RNA enzymes involved in monoamine metabolism, MAO-A, MAO-B and COMT, in the most brain areas studied.

### 5.2. Limitations

In our research, we did not analyze enzyme activity in the CNS samples, due to the peculiarities of the isolation method we used and the small volume of the resulting sample. Future data on the enzymatic activity of MAO-A, MAO-B and COMT could be a good addition to the study. In addition, data on the content of NE and analysis of NET mRNA in brain structures could extend our understanding of the processes occurring in the brain in hyperdopaminergic rats lacking DAT, which could be an important topic for further investigations.

*Level of 5-HIAA in the striatum was excluded from this article because the peak of 5-HIAA is superimposed by the peak of the extracellular metabolite DA—3-MT. Normally, it is very low in the striatum and this can be ignored, but in DAT-KO rats it is increased by five times [23].

## 6. Conclusions

**Figure 1:**
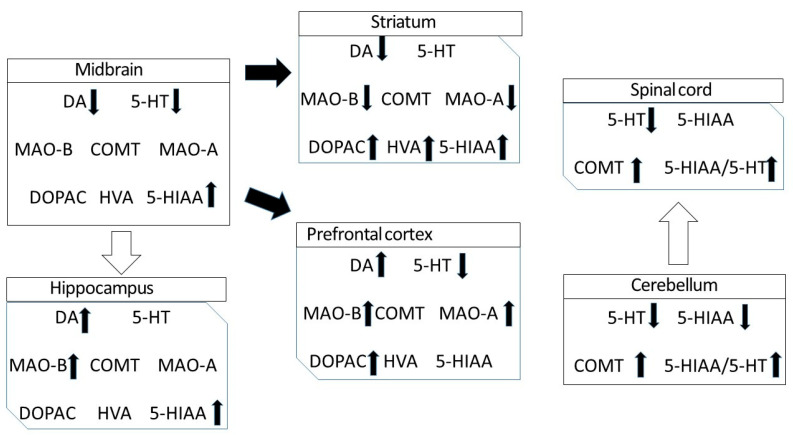
Neurochemical changes in various brain areas in hyperdopaminergic DAT-KO rats. Black arrows indicate dopaminergic pathways.

This study highlights the important role of DAT not only in the striatum but in other various structures in the brain, including the midbrain, frontal cortex and hippocampus, that remained essentially unexplored so far in DAT-KO animals. Furthermore, remarkable changes were observed in the regulation of serotonin system in most brain areas investigated, which was most pronounced in the cerebellum and spinal cord, thus demonstrating the close interaction of dopamine and serotonin systems in the brain. Furthermore, we have shown that *dat* gene knockout leads not only to a significant alteration in dopamine and serotonin neurotransmission in various structures of the CNS, but also to changes in levels of mRNA of monoamine-metabolizing enzymes. The results are summarized in Figure 1.

Finally, these data indicate that drugs that affect the dopaminergic system should certainly also affect the serotonergic system, and it is important to consider this possibility when predicting the possible therapeutic or side effects of drugs affecting the dopaminergic system.

## Figures and Tables

**Table 1:** Primers used for RT-PCR.

No.	Name	Primer Sequence	Annealing Temperature
1	MAO-A	Forward 5′-GCCAGGAACGGAAATTTGTA-3′; reverse 5′-TCTCAGGTGGAAGCTCTGGT-3′	65 °C
2	MAO-B	Forward 5′-TGGGCCAAGAGATTCCCAGTGATG-3′; reverse 5′-AGAGTGTGGCAATCTGCTTTGTAG-3′	60 °C
3	COMT130	Forward 5′-CTGGAGGCCATCGACACCTA-3′; reverse 5′-AGTAAGCTCCCAGCTCCAGCA-3′	60 °C
4	18S (housekeeping gene)	Forward 5′-ACGGACCAGAGCGAAAGCAT-3′; reverse 5′-TGTCAATCCTGTCCGTGTCC-3′	60 °C
5	PPI (housekeeping gene)	Forward 5′-GGATTTGGCTATAAGGGTTC-3′; reverse 5′-GTTGTCCACAGTCGGAGA-3′	60 °C

**Table 2:** Concentrations of monoamines in the different brain regions of DAT-KO rats, ng/mg protein.

	DA	DOPAC	HVA	5-HT	5-HIAA
Midbrain					
*DAT-WT*	5.1± 0.9	No data	No data	5.1 ± 0.0	0.1 ± 0.0
*DAT-KO*	2.8 ± 0.1	No data	No data	2.7 ± 0.1	0.3 ± 0.0
*t*	2.4			1.2	−5.4
*p*	0.012			0.008	0.003
Striatum					
*DAT-WT*	64.3 ± 2.7	6.0 ± 0.7	3.8 ± 0.7	8.2 ± 0.8	* excluded data
*DAT-KO*	7.5 ± 1.7	13.8 ± 1.7	13.8 ± 1.7	5.4 ± 1.3	* excluded data
*t*	18.9	−3.7	−5.3	1.7	
*p*	0.0002	0.0140	0.0075	0.1326	
Prefrontal cortex					
*DAT-WT*	0.8 ± 0.0	0.2 ± 0.0	0.3 ± 0.1	16.5 ± 2.7	13.1 ± 1.9
*DAT-KO*	1.8 ± 0.1	0.5 ± 0.1	0.3 ± 0.1	6.8 ± 0.7	9.7 ± 4.1
*t*	−16.0	−2.8	0.4	3.7	0.6
*p*	<0.0001	0.0271	0.6730	0.0069	0.5264
Hippocampus					
*DAT-WT*	0.4 ± 0.0	0.2 ± 0.0	No data	13.1 ± 0.7	15.5 ± 0.2
*DAT-KO*	0.9 ± 0.1	0.2 ± 0.0	No data	13.6 ± 0.5	17.0 ± 0.2
*t*	−5.0	0.1		−0.6	−4.3
*p*	0.0043	0.9513		0.5432	0.0078
Medulla oblongata					
*DAT-WT*	1.1 ± 0.2	0.3 ± 0.1	0.4 ± 0.1	17.5 ± 3.6	13.4 ± 2.3
*DAT-KO*	0.3 ± 0.1	0.1 ± 0.0	0.1 ± 0.0	0.1 ± 0.1	3.9 ± 1.1
*t*	3.9	4.4	2.1	5.5	4.0
*p*	0.0057	0.0032	0.0742	0.0009	0.0052
Cerebellum					
*DAT-WT*	1.0 ± 0.1	0.1 ± 0.0	0.1 ± 0.0	2.7 ± 0.9	1.6 ± 0.2
*DAT-KO*	0.7 ± 0.1	0.1 ± 0.0	0.1 ± 0.0	0.2 ± 0.0	0.8 ± 0.1
*t*	1.8	1.8	2.1	2.9	3.0
*p*	0.1229	0.1134	0.0742	0.0220	0.0191
Spinal cord					
*DAT-WT*	0.5 ± 0.1	0.2 ± 0.0	0.3 ± 0.1	6.0 ± 0.6	4.4 ± 0.8
*DAT-KO*	0.1 ± 0.0	0.1 ± 0.0	0.1 ± 0.1	1.8 ± 0.1	5.3 ± 0.9
*t*	3.4	1.4	1.1	7.9	−0.8
*p*	0.0120	0.1889	0.3025	0.0001	0.4747

* See in the limitations. DA—dopamine, DOPAC—3,4-dihydroxyphenylacetic acid, HVA—homovanillic acid, 5-HT—5-hydroxytryptamine (serotonin), 5-HIAA—5-hydroxyindoleacetic acid. The data are presented as mean ± SEM, *t*-test. DAT-KO (*n* = 13) and DAT-WT (*n* = 10).

**Table 3:** Turnover rates of DA and 5-HT in the different areas of DAT-KO rats, A.U.

	DOPAC/DA	HVA/DA	5-HIAA/5-HT
Striatum			
*DAT-WT*	0.1 ± 0.0	0.1 ± 0.0	No data
*DAT-KO*	2.0 ± 0.2	3.8 ± 0.4	No data
*t*	−9.7	−9.2	
*p*	0.0023	0.0027	
Prefrontal cortex			
*DAT-WT*	0.3 ± 0.1	0.4 ± 0.1	0.9 ± 0.2
*DAT-KO*	0.3 ± 0.0	0.1 ± 0.1	1.5 ± 0.6
*t*	0.2	2.4	−0.9
*p*	0.8200	0.0475	0.4201
Hippocampus			
*DAT-WT*	0.6 ± 0.1	No data	1.2 ± 0.0
*DAT-KO*	0.3 ± 0.0	No data	1.3 ± 0.0
*t*	4.3		−1.2
*p*	0.0075		0.2918
Medulla oblongata			
*DAT-WT*	0.3 ± 0.0	0.4 ± 0.1	0.9 ± 0.3
*DAT-KO*	0.2 ± 0.0	0.5 ± 0.1	0.0 ± 0.0
*t*	3.5	−1.3	
*p*	0.0094	0.2309	
Cerebellum			
*DAT-WT*	0.1 ± 0.0	0.1 ± 0.0	0.7 ± 0.2
*DAT-KO*	0.2 ± 0.1	0.2 ± 0.1	3.5 ± 0.3
*t*	−0.6	−0.9	−6.9
*p*	0.5745	0.3592	0.0002
Spinal cord			
*DAT-WT*	0.4 ± 0.2	0.7 ± 0.4	0.7 ± 0.1
*DAT-KO*	0.8 ± 0.1	0.6 ± 0.4	3.1 ± 0.7
*t*	−1.7	0.1	−2.8
*p*	0.1272	0.8975	0.0281

DA—dopamine, DOPAC—3,4-dihydroxyphenylacetic acid, HVA—homovanillic acid, 5-HT—5-hydroxytryptamine (serotonin), 5-HIAA—5-hydroxyindoleacetic acid. The data are presented as mean ± SEM, *t*-test. DAT-KO (*n* = 13) and DAT-WT (*n* = 10).

**Table 4:** Normalized relative gene expression of MAO-A, MAO-B and COMT mRNA in the different regions of DAT-KO rats, A.U.

	MAO-A	MAO-B	COMT
Striatum			
*DAT-WT*	1.0 ± 0.1	1.0 ± 0.1	1.0 ± 0.1
*DAT-KO*	0.4 ± 0.1	0.2 ± 0.1	1.0 ± 0.2
*t*	7.4	6.4	0.2
*p*	0.0003	0.0002	0.8688
Prefrontal Cortex			
*DAT-WT*	1.0 ± 0.1	1.0 ± 0.1	1.0 ± 0.1
*DAT-KO*	2.1 ± 0.2	2.0 ± 0.4	1.2 ± 0.2
*t*	−4.2	−2.3	−0.8
*p*	0.0031	0.0454	0.4283
Hippocampus			
*DAT-WT*	1.0 ± 0.1	1.0 ± 0.1	1.0 ± 0.1
*DAT-KO*	0.6 ± 0.2	2.2 ± 0.5	2.1 ± 0.7
*t*	1.8	−2.7	−1.6
*p*	0.1104	0.029	0.1561
Medulla oblongata			
*DAT-WT*	1.2 ± 0.2	1.0 ± 0.2	1.0 ± 0.1
*DAT-KO*	0.1 ± 0.1	0.1 ± 0.1	0.2 ± 0.1
*t*	5.8	3.9	4.5
*p*	0.0004	0.0048	0.0015
Cerebellum			
*DAT-WT*	0.1 ± 0.0	0.1 ± 0.0	0.7 ± 0.2
*DAT-KO*	0.2 ± 0.1	0.2 ± 0.1	3.5 ± 0.3
*t*	−0.6	−0.9	−6.9
*p*	0.5745	0.3592	0.0002
Spinal cord			
*DAT-WT*	1.1 ± 0.1	1.0 ± 0.1	1.0 ± 0.1
*DAT-KO*	0.9 ± 0.1	13.0 ± 2.0	10.8 ± 3.5
*t*	0.8	−5.8	−2.8
*p*	0.4301	0.0004	0.0240

The data are presented as mean ± SEM, *t*-test. DAT-KO (*n* = 13) and DAT-WT (*n* = 10).

## Data Availability

There are no publicly accessible archives, but we are happy to provide any primary data upon request.

## References

[1] Lerner T.N., Holloway A.L., Seiler J.L. (2020). Dopamine, Updated: Reward Prediction Error and Beyond. Curr. Opin. Neurobiol..

[2] Torrisi S.A., Laudani S., Contarini G., De Luca A., Geraci F., Managò F., Papaleo F., Salomone S., Drago F., Leggio G.M. (2020). Dopamine, Cognitive Impairments and Second-Generation Antipsychotics: From Mechanistic Advances to More Personalized Treatments. Pharmaceuticals.

[3] Franco R., Reyes-Resina I., Navarro G. (2021). Dopamine in Health and Disease: Much More Than a Neurotransmitter. Biomedicines.

[4] Bacqué-Cazenave J., Bharatiya R., Barrière G., Delbecque J.-P., Bouguiyoud N., Di Giovanni G., Cattaert D., De Deurwaerdère P. (2020). Serotonin in Animal Cognition and Behavior. Int. J. Mol. Sci..

[5] Bamalan O.A., Moore M.J., Khalili Y.A., Al Khalili Y. (2022). Physiology, Serotonin.

[6] Yagishita S. (2019). Transient and sustained effects of dopamine and serotonin signaling in motivation-related behavior. Psychiatry Clin. Neurosci..

[7] Weintraub D., Claassen D.O. (2017). Impulse Control and Related Disorders in Parkinson’s Disease. Int. Rev. Neurobiol..

[8] van Galen K.A., ter Horst K.W., Booij J., la Fleur S.E., Serlie M.J. (2018). The role of central dopamine and serotonin in human obesity: Lessons learned from molecular neuroimaging studies. Metabolism.

[9] Devroye C., Cathala A., Piazza P.V., Spampinato U. (2018). The central serotonin2B receptor as a new pharmacological target for the treatment of dopamine-related neuropsychiatric disorders: Rationale and current status of research. Pharmacol. Ther..

[10] Momiyama T., Nishijo T. (2017). Dopamine and Serotonin-Induced Modulation of GABAergic and Glutamatergic Transmission in the Striatum and Basal Forebrain. Front. Neuroanat..

[11] Hikosaka O., Sesack S.R., Lecourtier L., Shepard P.D. (2008). Habenula: Crossroad between the Basal Ganglia and the Limbic System. J. Neurosci..

[12] Schrag A., Taddei R.N. (2017). Depression and Anxiety in Parkinson’s Disease. Int. Rev. Neurobiol..

[13] Vo A., Ganjavi H., MacDonald P.A. (2018). Levodopa has mood-enhancing effects in healthy elderly adults. Int. J. Geriatr. Psychiatry.

[14] Baronti F., Davis T.L., Boldry R.C., Mouradian M.M., Chase T.N. (1992). Deprenyl effects on levodopa pharmacodynamics, mood, and free radical scavenging. Neurology.

[15] Metzger M., Souza R., Lima L.B., Bueno D., Gonçalves L., Sego C., Donato J., Shammah-Lagnado S.J. (2019). Habenular connections with the dopaminergic and serotonergic system and their role in stress-related psychiatric disorders. Eur. J. Neurosci..

[16] Vanmechelen I., Dan B., Feys H., Monbaliu E. (2019). Test–retest reliability of the Dyskinesia Impairment Scale: Measuring dystonia and choreoathetosis in dyskinetic cerebral palsy. Dev. Med. Child. Neurol..

[17] Seo D., Patrick C.J., Kennealy P.J. (2008). Role of Serotonin and Dopamine System Interactions in the Neurobiology of Impulsive Aggression and its Comorbidity with other Clinical Disorders. Aggress. Violent Behav..

[18] Kapur S., Remington G. (1996). Serotonin-dopamine interaction and its relevance to schizophrenia. Am. J. Psychiatry.

[19] Daw N.D., Kakade S., Dayan P. (2002). Opponent interactions between serotonin and dopamine. Neural Netw..

[20] Zhou F.M., Liang Y., Salas R., Zhang L., De Biasi M., Dani J.A. (2005). Corelease of dopamine and serotonin from striatal dopamine terminals. Neuron.

[21] Shi W.X., Nathaniel P., Bunney B.S. (1995). Ritanserin, a 5-HT2A/2C antagonist, reverses direct dopamine agonist-induced inhibition of midbrain dopamine neurons. J. Pharmacol. Exp. Ther..

[22] Larsen M.B., Sonders M.S., Mortensen O.V., Larson G.A., Zahniser N.R., Amara S.G. (2011). Dopamine transport by the serotonin transporter: A Mechanistically distinct mode of substrate translocation. J. Neurosci..

[23] Jones S.R., Gainetdinov R.R., Jaber M., Giros B., Wightman R.M., Caron M.G. (1998). Profound neuronal plasticity in response to inactivation of the dopamine transporter. Proc. Natl. Acad. Sci. USA.

[24] Leo D., Sukhanov I., Zoratto F., Illiano P., Caffino L., Sanna F., Messa G., Emanuele M., Esposito A., Dorofeikova M. (2018). Pronounced Hyperactivity, Cognitive Dysfunctions, and BDNF Dysregulation in Dopamine Transporter Knock-out Rats. J. Neurosci..

[25] Giros B., Jaber M., Jones S.R., Wightman R.M., Caron M.G. (1996). Hyperlocomotion and indifference to cocaine and amphetamine in mice lacking the dopamine transporter. Nature.

[26] Shen H.W., Hagino Y., Kobayashi H., Shinohara-Tanaka K., Ikeda K., Yamamoto H., Yamamoto T., Lesch K.P., Murphy D.L., Hall F.S. (2004). Regional Differences in Extracellular Dopamine and Serotonin Assessed by In Vivo Microdialysis in Mice Lacking Dopamine and/or Serotonin Transporters. Neuropsychopharmacology.

[27] Yamashita M., Fukushima S., Shen H.-w., Hall F.S., Uhl G.R., Numachi Y., Kobayashi H., Sora I. (2006). Norepinephrine Transporter Blockade can Normalize the Prepulse Inhibition Deficits Found in Dopamine Transporter Knockout Mice. Neuropsychopharmacology.

[28] Fox M.A., Panessiti M.G., Hall F.S., Uhl G.R., Murphy D.L. (2013). An evaluation of the serotonin system and perseverative, compulsive, stereotypical, and hyperactive behaviors in dopamine transporter (DAT) knockout mice. Psychopharmacology.

[29] Kurzina N., Aristova I., Volnova A., Gainetdinov R. (2020). Deficit in working memory and abnormal behavioral tactics in dopamine transporter knockout rats during training in the 8-arm maze. Behav. Brain Res..

[30] Paxinos G., Watson C. (2013). Rat Brain in Stereotaxic Coordinates.

[31] Jamal M., Ito A., Miki T., Suzuki S., Ohta K.-I., Kinoshita H. (2022). Ethanol concentration induces production of 3,4-dihydroxyphenylacetic acid and homovanillic acid in mouse brain through activation of monoamine oxidase pathway. Neurosci. Lett..

[32] Jaber M., Dumartin B., Sagné C., Haycock J.W., Roubert C., Giros B., Bloch B., Caron M.G. (1999). Differential regulation of tyrosine hydroxylase in the basal ganglia of mice lacking the dopamine transporter. Eur. J. Neurosci..

[33] Jones S.R., Bowman B.P., Kuhn C.M., Wightman R.M. (1996). Development of Dopamine Neurotransmission and Uptake Inhibition in the Caudate Nucleus as Measured by Fast-Cyclic Voltammetry. Synapse.

[34] Gainetdinov R.R., Caron M.G. (2003). Monoamine transporters: From genes to behavior. Annu. Rev. Pharmacol. Toxicol..

[35] Sotnikova T.D., Beaulieu J.M., Barak L.S., Wetsel W.C., Caron M.G., Gainetdinov R.R. (2005). Dopamine-independent locomotor actions of amphetamines in a novel acute mouse model of Parkinson disease. PLoS Biol..

[36] Sukhanov I., Dorotenko A., Fesenko Z., Savchenko A., Efimova E.V., Mor M.S., Belozertseva I.V., Sotnikova T.D., Gainetdinov R.R. (2022). Inhibition of PDE10A in a New Rat Model of Severe Dopamine Depletion Suggests New Approach to Non-Dopamine Parkinson’s Disease Therapy. Biomolecules.

[37] Volavka J., Bilder R., Nolan K. (2004). Catecholamines and aggression: The role of COMT and MAO polymorphisms. Ann. N. Y. Acad. Sci..

[38] Hao H., Shao M., An J., Chen C., Feng X., Xie S., Gu Z., Chan P. (2014). Association of Catechol-O-Methyltransferase and monoamine oxidase B gene polymorphisms with motor complications in parkinson’s disease in a Chinese population. Park. Relat. Disord..

[39] Salvatore M.F., Pruett B.S., Spann S.L., Dempsey C. (2009). Aging Reveals a Role for Nigral Tyrosine Hydroxylase ser31 Phosphorylation in Locomotor Activity Generation. PLoS ONE.

[40] Eshleman A.J., Carmolli M., Cumbay M., Martens C.R., Neve K.A., Janowsky A. (1999). Characteristics of drug interactions with recombinant biogenic amine transporters expressed in the same cell type. J. Pharmacol. Exp. Ther..

[41] Sesack S.R., Hawrylak V.A., Matus C., Guido M.A., Levey A.I. (1998). Dopamine Axon Varicosities in the Prelimbic Division of the Rat Prefrontal Cortex Exhibit Sparse Immunoreactivity for the Dopamine Transporter. J. Neurosci..

[42] Schroder E.A., Wang L., Wen Y., Callahan L.A.P., Supinski G.S. (2021). Skeletal muscle-specific calpastatin overexpression mitigates muscle weakness in aging and extends life span. J. Appl. Physiol..

[43] Madras B.K., Miller G.M., Fischman A.J. (2005). The Dopamine Transporter and Attention-Deficit/Hyperactivity Disorder. Biol. Psychiatry.

[44] Xu T.X., Sotnikova T.D., Liang C., Zhang J., Jung J.U., Spealman R.D., Gainetdinov R.R., Yao W.D. (2009). Hyperdopaminergic tone erodes prefrontal long-term potential via a D2 receptor-operated protein phosphatase gate. J. Neurosci..

[45] Turi G.F., Li W.K., Chavlis S., Pandi I., O’Hare J., Priestley J.B., Grosmark A.D., Liao Z., Ladow M., Zhang J.F. (2019). Vasoactive Intestinal Polypeptide-Expressing Interneurons in the Hippocampus Support Goal-Oriented Spatial Learning. Neuron.

[46] Adinolfi A., Zelli S., Leo D., Carbone C., Mus L., Illiano P., Alleva E., Gainetdinov R.R., Adriani W. (2019). Behavioral characterization of DAT-KO rats and evidence of asocial-like phenotypes in DAT-HET rats: The potential involvement of norepinephrine system. Behav. Brain Res..

[47] Mialet-Perez J., Santin Y., Parini A. (2018). Monoamine oxidase-A, serotonin and norepinephrine: Synergistic players in cardiac physiology and pathology. J. Neural Transm..

[48] Ito M. (2013). Error detection and representation in the olivo-cerebellar system. Front. Neural Circuits.

[49] Stoodley C.J. (2016). The cerebellum and neurodevelopmental disorders. Cerebellum.

[50] Sinzig J., Walter D., Doepfner M. (2009). Attention Deficit/Hyperactivity Disorder in Children and Adolescents with Autism Spectrum Disorder. J. Atten. Disord..

[51] Ronald A., Simonoff E., Kuntsi J., Asherson P., Plomin R. (2008). Evidence for overlapping genetic influences on autistic and ADHD behaviours in a community twin sample. J. Child. Psychol. Psychiatry.

[52] Bruchhage M.M.K., Bucci M.P., Becker E.B.E. (2018). Cerebellar involvement in autism and ADHD. Handb. Clin. Neurol..

[53] Nijmeijer K.J., Huijsman R., Fabbricotti I.N. (2014). Exploring the Role of Ownership Structures in the Results of Professional Health Care Franchises from a Multi-Actor Perspective. J. Mark. Channels.

[54] Saitow F., Hirono M., Suzuki H. (2013). Serotonin and synaptic transmission in the cerebellum. Handbook of the Cerebellum and Cerebellar Disorders.

[55] Chugani D.C., Muzik O., Behen M., Rothermel R., Janisse J.J., Lee J., Chugani H.T. (1999). Developmental Changes in Brain Serotonin Synthesis Capacity in Autistic and Nonautistic Children. Ann. Neurol..

